# Molecular Characteristics and Quantitative Proteomic Analysis of *Klebsiella pneumoniae* Strains with Carbapenem and Colistin Resistance

**DOI:** 10.3390/antibiotics11101341

**Published:** 2022-09-30

**Authors:** Ling Hao, Xiao Yang, Huiling Chen, Zexun Mo, Yujun Li, Shuquan Wei, Ziwen Zhao

**Affiliations:** 1 Department of Pulmonary and Critical Care Medicine, Guangzhou First People’s Hospital, Guangzhou 510180, China; 2 Department of Laboratory Medicine, Guangzhou First People’s Hospital, Guangzhou 510180, China

**Keywords:** *Klebsiella pneumoniae*, multidrug-resistant, extensively drug-resistant, colistin, carbapenem, quantitative proteomics, tandem mass tag

## Abstract

Carbapenem-resistant *Klebsiella pneumoniae* (CRKP) are usually multidrug resistant (MDR) and cause serious therapeutic problems. Colistin is a critical last-resort therapeutic option for MDR bacterial infections. However, increasing colistin use has led to the emergence of extensively drug-resistant (XDR) strains, raising a significant challenge for healthcare. In order to gain insight into the antibiotic resistance mechanisms of CRKP and identify potential drug targets, we compared the molecular characteristics and the proteomes among drug-sensitive (DS), MDR, and XDR *K. pneumoniae* strains. All drug-resistant isolates belonged to ST11, harboring *bla_KPC_* and hypervirulent genes. None of the plasmid-encoded *mcr* genes were detected in the colistin-resistant XDR strains. Through a tandem mass tag (TMT)-labeled proteomic technique, a total of 3531 proteins were identified in the current study. Compared to the DS strains, there were 247 differentially expressed proteins (DEPs) in the MDR strains and 346 DEPs in the XDR strains, respectively. Gene Ontology (GO) and Kyoto Encyclopedia of Genes and Genomes (KEGG) enrichment analysis revealed that a majority of the DEPs were involved in various metabolic pathways, which were beneficial to the evolution of drug resistance in *K. pneumoniae*. In addition, a total of 67 DEPs were identified between the MDR and XDR strains. KEGG enrichment and protein–protein interaction network analysis showed their participation in cationic antimicrobial peptide resistance and two-component systems. In conclusion, our results highlight the emergence of colistin-resistant and hypervirulent CRKP, which is a noticeable superbug. The DEPs identified in our study are of great significance for the exploration of effective control strategies against infections of CRKP.

## 1. Introduction

Carbapenem-resistant *Klebsiella pneumoniae* (CRKP) strains, which account for 70–90% of clinical carbapenem resistant Enterobacteriaceae (CRE) infections [1,2], pose urgent threats to global public health because they are usually multidrug resistant (MDR), and few antibiotics retain activity against them [3]. Infections caused by CRKP are associated with greater disease severity, more complications, and higher mortality [4]. Especially for severely ill patients, mortality could exceed 50% even with adequate antibiotic treatment [5]. Colistin is a critical last-resort antimicrobial for serious infections caused by CRKP in clinics [6]. Although several novel drugs and combinations have been considered in the therapy of MDR Gram-negative bacteria, colistin will still be considered as a fundamental companion drug for the treatment of CRE [7]. However, the increasing and suboptimal use of colistin has led to the emergence of colistin-resistant CRKP, which are identified as extensively drug-resistant (XDR) strains [8], raising a significant challenge for healthcare. Hence, additional knowledge about the mechanism of drug resistance in CRKP is urgently needed to aid in the development of more effective anti-infectious methodologies.

The precise mechanism of action of colistin remains unclear, but a number of models have been proposed. Studies have shown that lipid A, a lipid component of lipopolysaccharide (LPS) in the bacterial outer membrane (OM), is the primary target of colistin [6]. Colistin is a cationic antimicrobial peptide that binds lipid A and causes cell membrane leakage [6]. The interaction between colistin and lipid A is affected when lipid A is modified with 4-amino-4-deoxy-l-arabinose (L-Ara4N) or phosphoethanolamine (PEtN), thus resulting in colistin resistance [6,9]. There are two main mechanisms of modification of lipid A: chromosome-mediated regulation pathways and plasmid-mediated mobile-resistance genes (*mcr-1* to *mcr-10*) [10,11,12,13]. In chromosomes, modifications of lipid A with L-Ara4N or PEtN are achieved by the activities of the *arnBCADTEF* and *pmrCAB* operons, respectively [10,11]. Mobile *mcr* genes can facilitate resistant gene transfer from one strain to another and spread rapidly [12,13]. In addition, it has been reported that capsular polysaccharide and multidrug efflux pumps can also confer tolerance to colistin, although their impact is limited [11,14,15]. Currently, the steps toward the development new drugs cannot keep up with the pace at which resistance is acquired by *Klebsiella pneumoniae* (*K. pneumoniae*). Significant improvements in the therapeutic strategies for XDR bacterial infections are still very poor.

Proteomic analyses based on mass spectrometry and bioinformatics have provided an opportunity to combat antibiotic resistance and reveal unknown functional relationships [16]. In this context, the mining of specific proteins or proteome sets from clinically useful drug-resistant (DR) *K. pneumoniae* strains is very important. The tandem mass tag (TMT)-labeled quantitative proteomic approach is an in vitro labeling technology that can detect differentially abundant proteins from up to 16 different samples at the same time, so inconsistent conditions for the identification and separation of all samples can be avoided and more accurate quantitative results can be obtained [17]. In order to gain insight into the antibiotic resistance mechanisms of *K. pneumoniae* and identify potential drug targets, the TMT-labeled proteomic technique was used to compare the proteomes of drug-sensitive (DS), MDR, and XDR strains. Meanwhile, we also investigated the presence of *mcr* genes in colistin-resistant XDR strains. Such strains were also examined for the presence of carbapenemases and hypervirulent genes, sequence types (STs), hypermucoviscosity phenotypes, and their ability to form biofilms.

## 2. Results

### 2.1. Antimicrobial Susceptibility Profile and Molecular Characteristics of K. pneumoniae Isolates

The antimicrobial susceptibility profile showed that all DR isolates included in this study were resistant to 13 out of the 16 tested antibiotics. The MIC values for colistin of the XDR strains ranged from 32 to >64 μg/mL. The antibiotic susceptibility results for the nine isolates are summarized in Table 1.

All DR isolates were found to be positive for *bla_KPC_*. However, none of the plasmid-encoded *mcr* genes were detected in the colistin-resistant XDR strains. According to Multilocus Sequence Typing (MLST), all DR strains belonged to ST11 (Table 2). Hypervirulent genes *peg-344*, *iroB*, *iucA*, *_p_rmpA,* and *_p_rmpA2* were reported to be the biomarkers for defining hypervirulent *K. pneumoniae* (hvKP) (diagnostic accuracy >0.95), which is an evolving pathotype with more virulence than classical *K. pneumoniae,* and it infects healthy individuals in communities [18]. In our study, all DR isolates carried at least two hypervirulent genes, indicating the cooccurrence of drug resistance and hypervirulence in *K. pneumoniae* (Table 2). The MDR2 strain from ST11 harboring all five hypervirulent biomarkers might have the highest virulence potential. Interestingly, a string test showed that all MDR strains were positive and considered to have hypermucoviscosity, while all XDR strains were negative. In addition, the mean OD590 nm values obtained with the quantitative biofilm production assay are listed in Table 2. The XDR1 strain was a strong biofilm producer. The DS1, DS3, and MDR1 strains were weak biofilm producers, and the MDR3 strain showed no biofilm capabilities. The rest of the strains showed moderate biofilm-forming capacities. The results showed that the colistin-resistant XDR strains showed higher biofilm-forming capacities (0.59467 ± 0.14465) than the colistin-sensitive MDR strains (0.26778 ± 0.08842).

### 2.2. Statistics of the Spectrum Data of the Proteome

**Figure 1:**
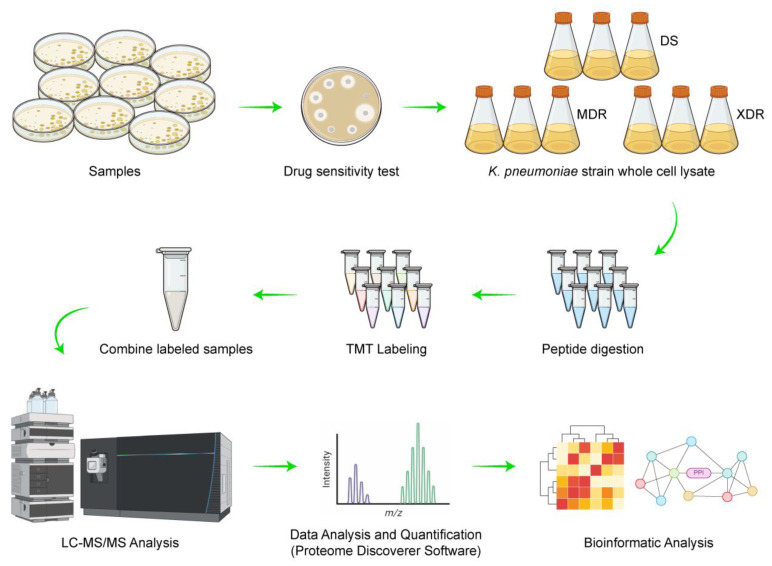
Experimental workflow of the quantitative proteomic analysis of DEPs in *K. pneumoniae*.

**Figure 2:**
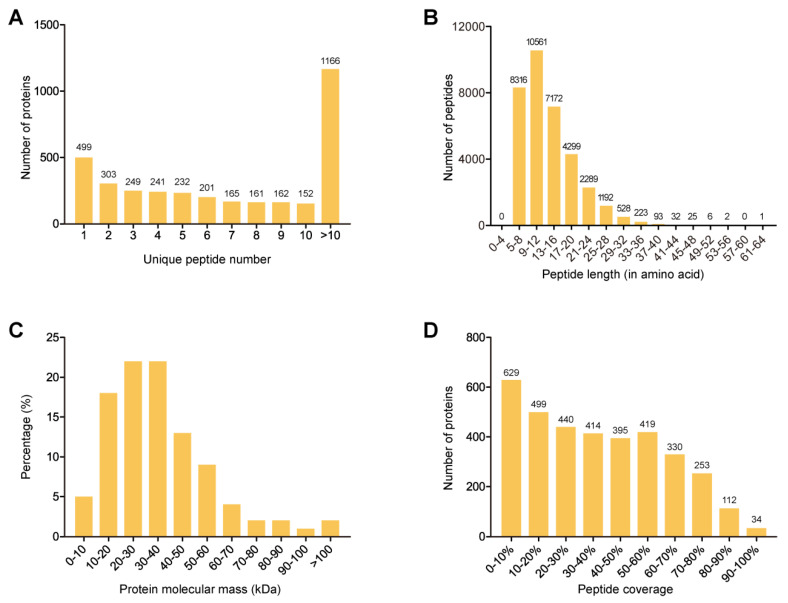
Profiles of the whole-cell-lysate proteins identified in nine *K. pneumoniae* isolates. (**A**) Quantitative distribution of unique peptides identified for individual proteins. (**B**) Length distribution of the identified peptides. (**C**) Distribution of molecular mass among all identified proteins. (**D**) Proportional distribution of the amino acid sequence of each identified protein covered by peptides.

In total, there were three groups in our experimental design, and TMT-labeling was employed for protein quantification (Figure 1). LC-MS/MS analysis of the whole-cell-lysate proteins (extracted at 4 °C) from nine *K. pneumoniae* isolates generated a total of 513,635 raw spectra. With an FDR of <1% for protein and peptide identification, we obtained 96,134 qualified spectra, including 95,529 unique spectra, which were associated with 34,739 peptides (34,548 unique peptides). These peptides were mapped to 3531 proteins with at least one unique peptide per protein; there were at least two unique peptides for 3032 (85.9%) of the 3531 proteins identified (Figure 2A). The distribution of the peptide length is shown in Figure 2B. A total of 87.4% of the peptides had 5 to 20 amino acids after enzymatic hydrolysis, which indicated that the enzymatic hydrolysis was sufficient and the identification results were reliable. The molecular masses of most proteins were from 10 to 60 kDa, and 2% of the proteins with molecular masses >100 kDa were identified (Figure 2C). The identification was more convincing with the expansion of the peptide coverage distribution. In this study, 82.2% of the identified proteins had a peptide coverage of more than 10% (Figure 2D).

### 2.3. Identification of Differentially Expressed Proteins (DEPs) for Each Compared Group

**Figure 3:**
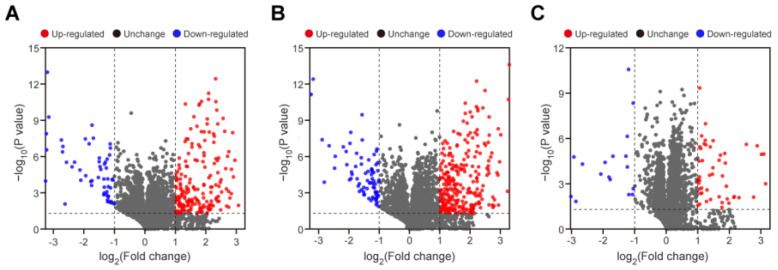
Volcano plots of the DEPs for each compared group. (**A**–**C**) Volcano plots for the screening of the DEPs in the MDR vs. DS strains (**A**), in the XDR vs. DS strains (**B**), and in the XDR vs. MDR strains (**C**). The dashed line represents the applied threshold (*p*-value < 0.05, fold change >2). The up-regulated and down-regulated proteins are shown in red and blue, respectively.

Based on the quantitative spectrum data, significantly, the DEPs between each compared group were determined with a fold change of >2 and a *p*-value < 0.05 (Figure 3). When compared to the DS strains, there were a total of 247 DEPs, including 194 up-regulated and 53 down-regulated proteins, in the MDR strains (Figure 3A). A total of 346 DEPs were identified between the XDR and DS strains, among which 270 proteins were significantly increased, while 76 proteins were decreased in the XDR strains (Figure 3B). In addition, there were a total of 67 DEPs, including 49 up-regulated and 18 down-regulated proteins, between the XDR and MDR strains (Figure 3C). The details of the DEPs in the MDR vs. DS strains, the XDR vs. DS strains, and the MDR vs. XDR strains are shown in Tables S1–S3.

### 2.4. Prediction of the Protein Functions of the DEPs between the MDR and DS Strains

**Figure 4:**
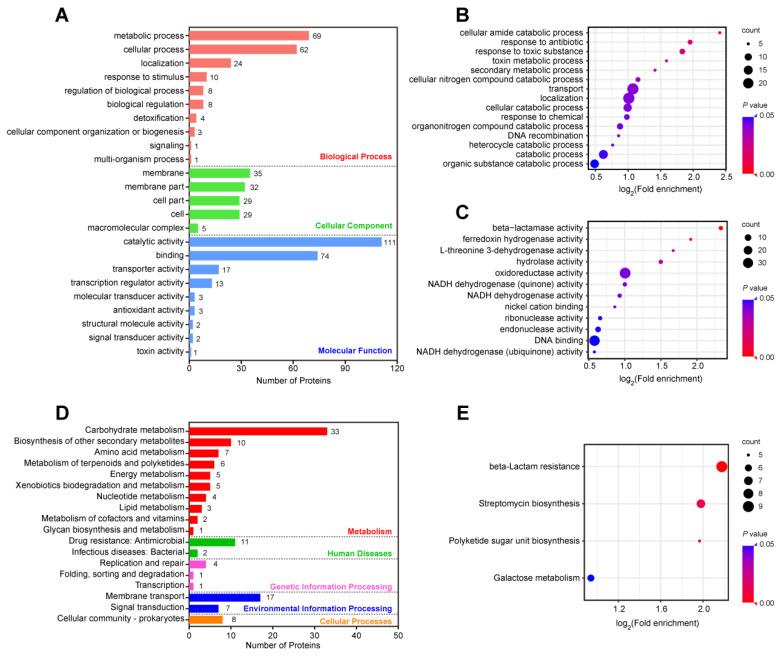
Functional and pathway enrichment analysis of the DEPs between the MDR and DS strains. (**A**) Numbers of the 247 DEPs in the biological process, cellular component, and molecular function categories revealed by the GO annotations. (**B**,**C**) GO functional enrichment analysis of biological processes (**B**) and molecular functions (**C**). (**D**) The KEGG pathway analysis of the 247 DEPs. (**E**) The enrichment analysis of the KEGG pathway.

The Gene Ontology (GO) classification of the 247 DEPs between the MDR and DS strains resulted in three broad categories (biological processes, cellular components, and molecular functions) and 24 GO terms (Figure 4A). In biological process category, 69 DEPs were associated with metabolic processes, 62 DEPs with cellular processes, and 24 DEPs with localization. In the cellular component category, 35 DEPs were detected in the membrane, 32 DEPs in the membrane part, and 29 DEPs in the cell part. In addition, the molecular functions associated with the DEPs were as follows: 111 DEPs were involved in catalytic activity, 74 DEPs were involved in binding, and 17 DEPs were detected mainly in transporter activity (Table S4). The functional enrichment analysis further determined the significantly enriched GO classification of the DEPs according to the *P*-values. The results indicated that the biological processes of the DEPs were related to transport, localization, and catabolic processes (Figure 4B). The molecular functions of the DEPs were concentrated on oxidoreductase activity, DNA binding, and endonuclease activity (Figure 4C).

Based on the Kyoto Encyclopedia of Genes and Genomes (KEGG) analysis, the DEPs were classified and annotated into metabolism, human diseases, genetic information processing, environmental information processing, and cellular processes (Figure 4D). Carbohydrate metabolism, biosynthesis of other secondary metabolites, and amino acid metabolism were the most common annotations of DEPs in the metabolism category, with 33, 10, and 7 proteins, respectively. Importantly, 11 DEPs, including AcrB, WecH, Bm3R1, SapB, OppC, OppA, OppF, and 4 β-lactamases (KPC-2, CTX-M-14, SHV-11, TEM-1), were enriched in drug resistance (Table S5). The pathway enrichment analysis showed that the DEPs were linked to beta-lactam resistance, streptomycin biosynthesis, polyketide sugar unit biosynthesis, and galactose metabolism (Figure 4E).

### 2.5. Prediction of the Protein Functions of the DEPs between the XDR and DS Strains

**Figure 5:**
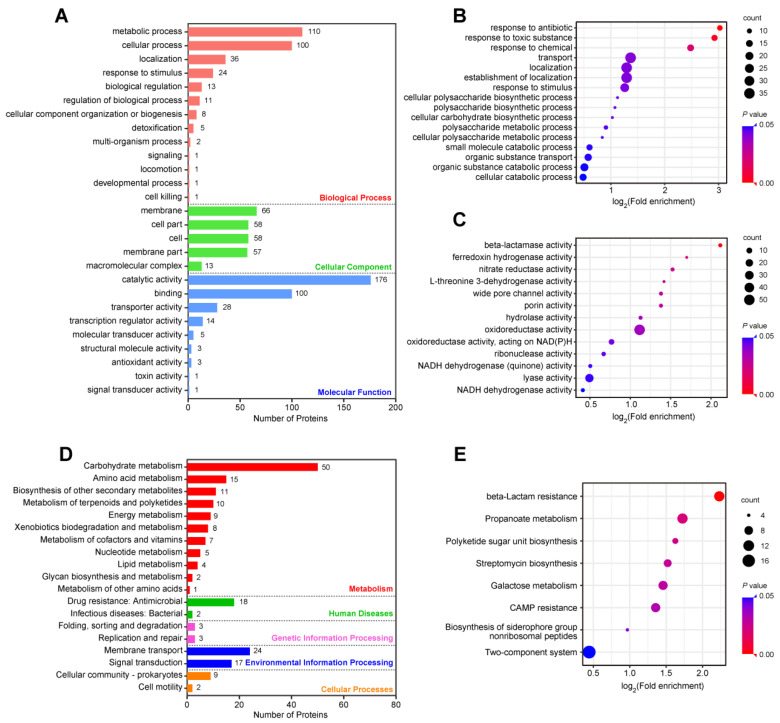
Functional and pathway enrichment analysis of the DEPs between the XDR and DS strains. (**A**) Numbers of the 346 DEPs in the biological process, cellular component, and molecular function categories revealed by the GO annotations. (**B**,**C**) GO functional enrichment analysis of biological processes (**B**) and molecular functions (**C**). (**D**) The KEGG pathway analysis of the 346 DEPs. (**E**) The enrichment analysis of the KEGG pathway.

The effects of the 346 DEPs between the XDR and DS strains on cellular processes were clarified using GO annotation and KEGG pathway analysis (Figure 5). The GO annotation of the 346 DEPs resulted in 27 GO terms (Figure 5A). The numbers of DEPs involved in biological process were as follows: 110 proteins were associated with metabolic processes, 100 proteins with cellular processes, and 36 proteins with localization. The DEPs in cellular components were mainly concentrated in the membrane (66 proteins), cell part (58 proteins), and cell (58 proteins). In addition, the top three items of molecular functions associated with DEPs were catalytic activity (176 proteins), binding (100 proteins), and transporter activity (28 proteins) (Table S6). The enrichment analysis of the biological processes showed that the DEPs were related to transport, localization, and establishment of localization (Figure 5B). In the molecular functions, the DEPs were associated with oxidoreductase activity and lyase activity (Figure 5C).

The KEGG pathway analysis for the DEPs between the XDR and DS strains showed that carbohydrate metabolism, amino acid metabolism, and biosynthesis of other secondary metabolites were still the most commonly annotated DEPs in the metabolism category (Figure 5D). Importantly, 18 DEPs, including AcrA, AcrB, WecH, Bm3R1, OppC, OppA, OppF, DegP, PmrD, 4 β-lactamases (KPC-2, CTX-M-14, SHV-11, TEM-1), and five enzymes that are responsible for lipid A modification (ArnB, ArnC, ArnA, ArnD, ArnT), were enriched in drug resistance (Table S7). The pathway enrichment analysis showed that the DEPs were linked to two-component systems (TCSs), beta-lactam resistance, propanoate metabolism, galactose metabolism, and cationic antimicrobial peptide (CAMP) resistance (Figure 5E).

### 2.6. Prediction of the Protein Functions of the DEPs between the MDR and XDR Strains

**Figure 6:**
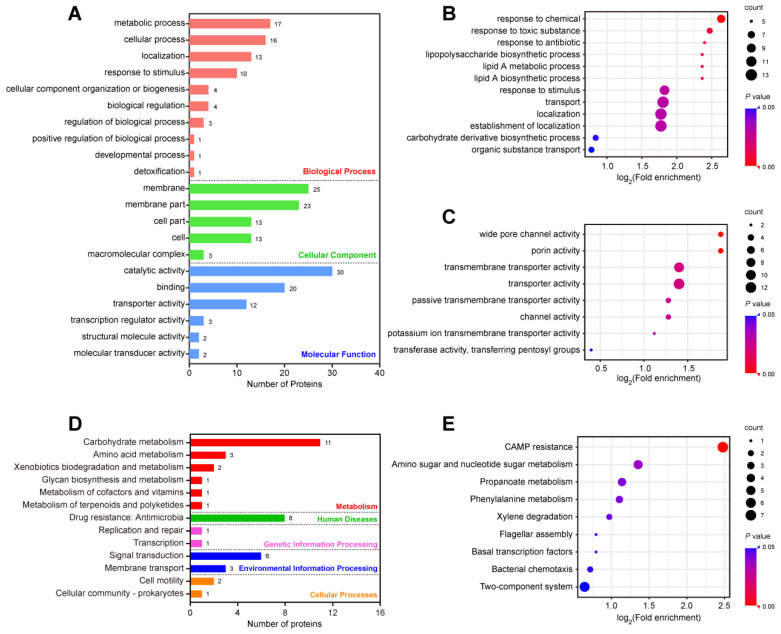
Functional and pathway enrichment analysis of the DEPs between the MDR and XDR strains. (**A**) Numbers of the 67 DEPs in the biological process, cellular component, and molecular function categories revealed by the GO annotations. (**B**,**C**) GO functional enrichment analysis of biological processes (**B**) and molecular functions (**C**). (**D**) The KEGG pathway analysis of the 67 DEPs. (**E**) The enrichment analysis of the KEGG pathway.

We further analyzed the effects of the DEPs between the XDR and MDR strains, which may offer an opportunity to find the potential mechanism of colistin resistance in *K. pneumoniae*. In the GO analysis of the 67 DEPs, there were 10, 5, and 6 enriched GO terms in the biological process, cellular component, and molecular function categories, respectively (Figure 6A). The most abundant GO terms included metabolic processes (17 proteins) in the biological process category, membrane (25 proteins) and membrane part (23 proteins) in the cellular component category, and catalytic activity (30 proteins) in the molecular function category (Table S8). The enrichment analysis of the biological process showed that the DEPs were related to transport, localization, and establishment of localization (Figure 6B). In molecular function, the DEPs were associated with transporter activity and transmembrane transporter activity (Figure 6C).

The KEGG pathway analysis for the DEPs between the MDR and XDR strains showed that most DEPs were annotated in carbohydrate metabolism in the metabolism category. In particular, eight proteins (ArnT, ArnD, ArnA, ArnC, ArnB, PmrD, YddW, OmpK36) were enriched in drug resistance (Table S9). The pathway enrichment analysis showed that a majority of the DEPs were involved in TCS, CAMP resistance, amino sugar and nucleotide sugar metabolism, propanoate metabolism, and phenylalanine metabolism.

**Figure 7:**
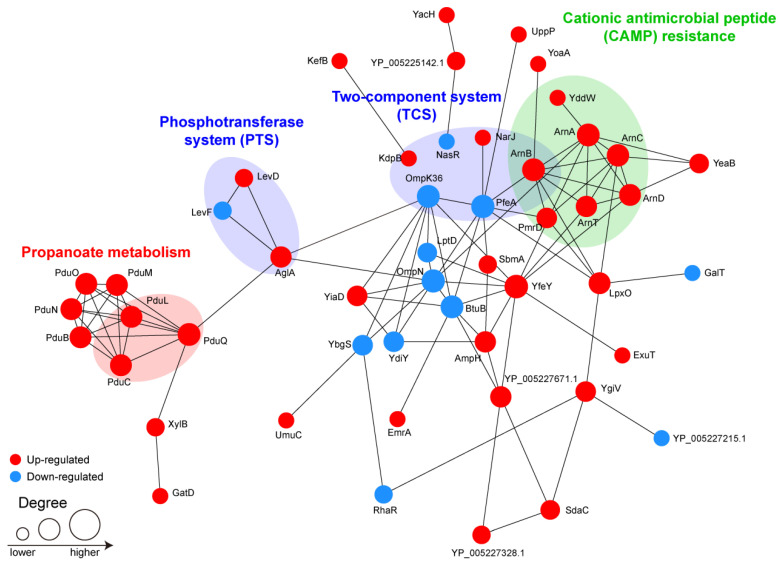
The interaction network analysis of the DEPs between the MDR and XDR strains. The size of the node represents the number of interacting proteins. The up-regulated proteins are marked in red, while the down-regulated proteins are marked in blue.

The 67 DEPs were mapped onto the STRING database, and 50 of them were retained to build a protein–protein interaction (PPI) network (Figure 7). Proteins that did not play roles in the construction of the network were filtered out. In particular, PfeA, OmpN, and YfeY, which had the largest number of interacting proteins, were the hubs of the network. The closely interacting DEPs were mainly distributed in four modules, namely, propanoate metabolism, phosphotransferase system (PTS), TCSs, and CAMP resistance.

## 3. Discussion

Nowadays, *K. pneumoniae* has become a significant clinical concern in nosocomial environments worldwide because of its remarkable abilities for drug resistance [1,2]. Worse still, a combination of drug-resistant and hypervirulent genes in *K. pneumoniae* strains could exacerbate the scarcity of effective treatments, resulting in related fatal hospital outbreaks [19,20]. A comparative analysis of molecular characteristics and proteomic changes from clinical *K. pneumoniae* isolates allowed us to reveal the unknown mechanism of antibiotic resistance of this bacterial pathogen. In the present study, antibiotic resistance, virulence-associated genes, STs, hypermucoviscosity phenotypes, biofilm-forming ability, and the proteomes among DS, MDR, and XDR *K. pneumoniae* strains were investigated. All DR strains belonged to ST11, harboring *bla_KPC_* and hypervirulent genes. None of the plasmid-encoded *mcr* genes were detected in the colistin-resistant XDR strains. The string test showed that all MDR strains were positive and considered to display hypermucoviscosity, while the XDR strains were negative. The biofilm formation capacity of the colistin-resistant XDR strains was higher than that of the colistin-sensitive MDR strains. Through the TMT-labeled quantitative proteomic technique, we identified a total of 34,548 unique peptides associated with 3531 proteins among the DS, MDR, and XDR strains. Compared to the DS strains, there were 247 DEPs in the MDR strains and 346 DEPs in the XDR strains. The GO and KEGG enrichment analysis revealed that various metabolic pathways in which the DEPs participated were conducive to the evolution of DS strains into MDR and XDR strains. In addition, a total of 67 DEPs were identified between the MDR and XDR strains, of which 49 were up-regulated and 18 were down-regulated. Notably, the DEPs related to drug resistance were ArnT, ArnD, ArnA, ArnC, ArnB, PmrD, YddW, and OmpK36. Bioinformatic studies such as KEGG enrichment and PPI network analysis showed their participation in CAMP resistance and TCSs. Therefore, these DEPs are of great significance for exploring effective control strategies against infections of CRKP.

Under antibiotic pressure, *K. pneumoniae* can rapidly evolve and develop resistance, showing alterations in protein expressions, responsive signals, and cellular processes [21]. In our report, there were 247 DEPs identified in the MDR strains and 346 DEPs identified in the XDR strains when compared to the DS strains. Notably, four β-lactamases, namely, KPC-2, CTX-M-14, SHV-11, and TEM-1, were detected in all DR strains, and these comprise the major mechanism of the resistance of *K. pneumoniae* to most β-lactam antibiotics. The expression of efflux pumps in order to pump out an antibiotic is another common resistance mechanism for many antibiotics. In our study, KexD, AcrA, and AcrB were up-regulated in the DR strains compared to the DS strains. KexD is a component of an energy-dependent efflux pump that belongs to the resistance nodulation division (RND) superfamily [22]. It could interact with other RND efflux components (e.g., the periplasmic protein AcrA and the porin TolC) to efflux antibiotics, such as novobiocin or erythromycin, and it was induced by *crrB* mutation to result in colistin resistance [23]. AcrA and ArcB are the components of an archetypal RND multidrug efflux pump, AcrAB-TolC, which has a critical role in resistance to multiple antibiotics, especially fluoroquinolones, tetracycline, and beta-lactam antibiotics, in MDR strains of *K. pneumoniae* [24,25]. In addition, WecH, Bm3R1, OppC, OppA, and OppF were the same DEPs in the MDR and XDR strains compared to the DS strains, and they were enriched in drug resistance. WecH is an O-acetyltransferase that is responsible for the incorporation of O-acetyl groups into the enterobacterial common antigen (ECA) trisaccharide repeat units [26]. Bm3R1 is a TetR family transcriptional regulator that acts as a chemical sensor to monitor the cellular environmental dynamics, which mediate an adaptive response to toxic fatty acids in *Bacillus megaterium* [27,28]. OppC, OppA, and OppF are oligopeptide permeases that have been proved to play a variety of important roles in nutrition and virulence in several bacteria [29]. Low expression of OppC, OppA, and OppF was associated with β-lactam resistance in *Streptococcus agalactiae* [30]. Importantly, bacterial metabolism plays a crucial role in mediating the cellular responses to antibiotic treatment [31]. Through the GO and KEGG pathway analysis, a majority of the DEPs in the DR vs. DS strains were involved in several metabolic processes, such as carbohydrate metabolism, amino acid metabolism, metabolism of terpenoids and polyketides, and galactose metabolism. Studies have shown that metabolism of carbohydrates, starches, sucrose, arginine, and its derivative, proline, played an important role in biofilm formation and maintenance [32,33,34]. Biofilms are communities of microorganisms that adhere to a surface, and they play a significant role in persistent bacterial infections [31]. Compared with planktonic bacteria, the resistance of bacteria within a biofilm to antibiotics is several orders of magnitude higher. Biofilm-forming ability has been associated with antibiotic resistance in *K. pneumoniae* [35,36]. Consistently with these studies [35,36], we found that the colistin-resistant XDR strains could have a strong biofilm-forming ability, which might make colonized *K. pneumoniae* more difficult to treat.

More importantly, we further identified 67 DEPs between the MDR and XDR strains and found that ArnT, ArnD, ArnA, ArnC, ArnB, PmrD, YddW, and OmpK36 were enriched in drug resistance. It is widely approved that colistin destroys the bacterial OM by binding to lipid A of LPS, leading to osmotic imbalance and cell death [6]. Alterations in LPS helped *K. pneumoniae* survive under colistin [6]. ArnT, ArnD, ArnA, ArnC, and ArnB, the gene products of the *arnBCADTEF* operon, are the enzymes responsible for the biosynthesis and addition of L-Ara4N to lipid A, which reduces the negative charge carried by LPS, causing a decreased ability of colistin to combine with bacteria [6]. Constitutive expression of the *arnBCADTEF* operon is achieved by the mutations in TCSs, mainly PmrA/PmrB and PhoP/PhoQ, leading to colistin resistance [10,11,23]. PmrD is a connector protein that conveys feedback between the PmrA/PmrB and PhoP/PhoQ TCSs [11,37]. Expression of *pmrD* in *K. pneumoniae* was dependent on PhoP; the activated PmrD would then bind to PmrA to inhibit its dephosphorylation and, eventually, turn on the expression of the *arnBCADTEF* operon for the resistance to colistin [11]. YddW is a divisome-localized glycosyl hydrolase that cleaves peptide-free peptidoglycan glycans in the OM layer, promoting OM constriction during cell division [38]. The overexpression of *yddW* alleviates the phenotypes of *Escherichia coli* (*E. coli*) in a defective Tol-Pal system, a multiprotein system in the OM of Gram-negative bacteria [38]. Inactivation of this system compromises the OM layer, resulting in hypersensitivity to many antibiotics [38]. In addition, porin loss could contribute to phenotypes that are resistant against antibiotics such as β-lactams [39]. Studies have shown that defects in *K. pneumoniae* porins OmpK35 and OmpK36 (the *E. coli* homologs are OmpF and OmpC, respectively) could lead to reduced sensitivity to carbapenems [40,41]. Substitutions in the *ompC* gene mediated carbapenem resistance among *Enterobacteriaceae* and were partly involved in colistin resistance [42]. In line with these studies [39,40,41], our proteomic data also showed that OmpK36 was decreased in the XDR strains compared to the MDR strains. In addition, we found that OmpN was also down-regulated in the colistin-resistant XDR strains. The overexpression of OmpN enhanced the sensitivity of *K. pneumoniae* to several antibiotics, such as cefuroxime, cefuroxime axetil, nitrofurantoin, and ampicillin/sulbactam [43], indicating that the decrease in OmpN might be responsible for drug resistance. However, the regulatory effect of OmpN on colistin resistance is unknown. Moreover, the KEGG pathway analysis and the PPI network showed that the DEPs between the MDR and XDR strains were mainly enriched in CAMP resistance and TCSs. In the CAMP resistance pathway, ArnBCADT, PmrD, and YddW were highly expressed in the colistin-resistant XDR strains, which indicated that lipid A modification is still the main mechanism of colistin resistance in *K. pneumoniae*. TCSs, which are typically composed of two proteins (a sensor kinase and a response regulator), are crucial for bacteria to maintain homeostasis, and they effectively sense and respond to environmental changes, such as nutrition, osmotic pressure, and antibiotic exposure [44]. In the current study, KdpB, OmpK36, PfeA, NasR, NarJ, and ArnB were involved in the TCS pathway. KdpB is a subunit of potassium-transporting ATPase [45]. The KdpATPase is an inducible high-affinity K^+^ transporter that maintains the desired concentration of internal K^+^ in bacterial cells [45]. The porin protein OmpK36 is regulated by the EnvZ/OmpR TCS and plays an important role in iron homeostasis [46]. PfeA is a ferric enterobactin receptor inducible by enterobactin under the conditions of iron limitation, which is regulated by a TCS PfeR/PfeS [47]. NasR and a TCS NARL/NARP regulate the synthesis of enzymes for nitrate/nitrite respiration and assimilation [48]. NarJ is a system-specific chaperone required for the formation of the respiratory nitrate reductase complex in *Escherichia coli* [49]. ArnB is the promoter region of the *arn* operon, which binds to the PhoP/PhoQ response regulator and regulates the modification of lipid A [50]. Studies have shown that multiple pathways might operate together to cause drug resistance, such as a cross-talk between the PhoP/PhoQ TCS and the Rcs system (regulator capsule synthesis) [51], as well as a cross-regulatory interaction between a specific CroS/CroR TCS and the HPr protein of PTS [52]. Future work will focus on understanding the detailed roles of these pathways in colistin resistance in order to make the bacteria sensitive.

In conclusion, our data highlight that the co-existence of drug-resistant and hypervirulent genes, as well as the strong biofilm-forming ability, will cause colistin-resistant CRKP to emerge as super-bugs. The proteomic results obtained the DEPs of DR strains, providing insights for understanding the mechanism of resistance to colistin in *K. pneumoniae*. Functional and pathway enrichment analyses of these DEPs laid a foundation for establishing effective control strategies against infections of CRKP.

## 4. Materials and Methods

### 4.1. Bacterial Strains and Culture

Nine strains of *K. pneumoniae* were isolated from the clinical samples of inpatients in our hospital. Among these strains, three (DS1, DS2, and DS3) were sensitive to all tested drugs, three (MDR1, MDR2, and MDR3) were CRKP strains that were susceptible to colistin, and the other three (XDR1, XDR2, and XDR3) were colistin-resistant CRKP strains. The XDR strains were matched with the MDR strains from the same patient obtained before the initiation of colistin treatment. All strains were identified using biochemical tests and the IVD MALDI Biotype system. Bacterial cells were cultured at 37 °C overnight on LuriaBertani (LB) plates (Difco, Sparks, MD). Then, a single colony was selected and inoculated under different culture conditions: The DS strains were inoculated in LB broth with no antibiotic, while the DR strains were cultured in LB broth supplemented with meropenem (16 µg/mL) and imipenem (16 µg/mL) and with or without colistin (4 µg/mL). The cultures were grown at 37 °C with shaking at 180 rpm until the OD_600_ reached 0.4–0.6 (mid-log growth phase), washed twice with cold PBS, and harvested for proteomic analysis.

### 4.2. Drug Sensitivity Test of Bacterial Strains

Routine antimicrobial susceptibility testing was performed with the Vitek-2 Compact automatic microbiology system (bioMerieux, Inc., Durham, NC, USA). The minimum inhibitory concentrations (MICs) were interpreted according to the breakpoints defined by the Clinical and Laboratory Standards Institute (CLSI), as described previously [53]. Susceptibility testing for colistin was further confirmed for all strains with the microdilution method outlined by the CLSI [54]. *E. coli* ATCC 25922 and *Pseudomonas aeruginosa* ATCC 27853 were used as quality control organisms for the antimicrobial susceptibility testing.

### 4.3. Identification of mcr, Carbapenemases, and Hypervirulent Genes

The genomic DNA of each isolate was extracted using a DNA extraction kit (Tiangen, Beijing, China) according to the manufacturer’s protocol. The presence of antibiotic resistance genes, including *mcr* genes (*mcr-1* to *mcr-8*) and carbapenemases (*bla_KPC_*, *bla_NDM_*, *bla_VIM_*, *bla_IMP_*, and *bla_OXA-48_* type), was analyzed with conventional PCR using validated primers and methods, and the amplified products were confirmed through sequencing [55,56]. The frequency of five hypervirulent genes, namely, *iucA*, *iroB*, *peg-344*, *_p_rmpA*, and *_p_rmpA2,* was determined through PCR with established primers and methods [18]. All analyses were performed with corresponding positive controls. All primer sequences are listed in Table S10.

### 4.4. Multilocus Sequence Typing (MLST)

MLST was performed on all *K. pneumoniae* isolates by amplifying and sequencing the seven standard housekeeping genes (*gapA*, *infB*, *mdh*, *pgi*, *phoE*, *rpoB*, and *tonB*), as described previously [57]. STs were assigned using the online database on the Pasteur Institute MLST website (http://bigsdb.pasteur.fr/klebsiella/klebsiella.html, accessed on 28 July 2022).

### 4.5. Detection of the Hypermucoviscosity Phenotype

A string test was used to detect the hypermucoviscosity phenotype, as described previously [58]. Briefly, an inoculation loop was used to stretch the bacterial colonies of *K. pneumoniae* isolates on Columbia blood agar plates (Crmicrobio, Jiangmen, China) from an overnight culture. Strains with the formation of viscous strings that were >5 mm in length were considered to be positive for the string test.

### 4.6. Biofilm Assay

Biofilm formation was determined by using the crystal violet staining method from the previous literature with little modification [59,60]. Briefy, 100 µL of overnight-grown culture with an optical density of OD_600_ = 0.1 was added into a 96-well plate. Sterile LB broth served as a negative control. After static incubation at 37 °C for 24 h, each well was washed gently with sterile PBS three times to remove the planktonic cells. After air drying, biofilms were stained with 1% (*w*/*v*) crystal violet for 15 min and then washed again with sterile PBS three times. Finally, the dye bound to adherent biomass was dissolved with 200 µL of absolute ethanol. Absorbance was measured using an automated microplate reader (Thermo Scientific, Waltham, MA, USA) at 590 nm. The tests were performed in triplicate, and the results were averaged. The strains were classified into non-biofilm producers, weak biofilm producers, moderate biofilm producers, and strong biofilm producers according to a previous report [35].

### 4.7. Whole-Cell-Lysate Protein Extraction from Bacterial Strains

The cell pellets were suspended in 500 µL of lysis buffer (4% SDS, 7 M urea, 2 M thiourea, 20 mM Tris-HCl, proteinase inhibitor cocktail, PH = 8.0) and lysed through ultrasonic decomposition in an ice bath at a speed of 60 Hz for 2 min. The supernatants were collected by centrifugation at 25,000× *g* at 4 °C for 15 min, reacted with 10 mM DTT (1,4-Dithiothreitol) for 30 min at 37 °C, and alkylated with 55 mM IAA (iodoacetamide) for another 45 min at room temperature in the dark. Then, the soluble proteins were precipitated for 2 h at −20 °C by adding cold acetone to the protein solution in excess (1:5 in volume). The precipitated proteins were collected by centrifugation (25,000× *g*, 4 °C, 20 min), air-dried, and then dissolved in an appropriate volume of lysis buffer (without SDS). Samples were further treated with ultrasound (60 Hz, 2 min) in an ice bath to promote protein dissolution. The collected whole-cell-lysate proteins were purified through ultracentrifugation at 25,000× *g* for 15 min at 4 °C. The protein concentrations were determined using the Bradford assay. The lysate proteins were identified with 12% SDS-PAGE.

### 4.8. Peptide Digestion and TMT Labeling

Peptide digestion was performed by following a previously published protocol [61]. Briefly, 100 µg of each lysate protein sample was first diluted in an appropriate volume of 0.5 M triethylamine borane (TEAB, Sigma-Aldrich, St Louis, MO, USA) to make a final concentration of <2 M urea, and then digested with 5 µg of trypsin at 37 °C for 4 h. The obtained peptide digests were desalted using a Strata-X column (Phenomenex, Torrance, CA, United States) and freeze-dried after salt removal. Then, 100 μg of dried peptide fragments were dissolved in 0.1 M TEAB (Sigma) to a final concentration of 3.74 µg/µL and mixed with different TMT isotopes at room temperature for 2 h (Table S11). The labeled peptides were pooled together for LC-MS/MS analysis.

### 4.9. LC-MS/MS Analysis of the Labeled Peptides

A total of 20 µg of pooled peptides were dried and then dissolved in 2 mL of Buffer A (5% acetonitrile, pH 9.8). Fractionation was carried out in an HPLC system (LC-20AD, Shimadzu, Kyoto, Japan) using a Gemini C18 column containing 5 µm particles (Phenomenex, Torrance, CA, USA). A total of 20 fractions were collected at a flow rate of 1 mL/min with Buffer B (95% acetonitrile, pH 9.8) with a multi-step gradient system as follows: 5% for 10 min, 5–35% for 40 min, 35–95% for 1 min, 95% for 3 min, and 5% for 10 min. Then, the fractions were freeze-dried, re-dissolved in Solvent A (2% acetonitrile, 0.1% formic acid), and centrifuged at 20,000× *g* for 10 min. The supernatant was applied to an EASY-nLC^TM^ 1200 system (Thermo Fisher Scientific, San Jose, CA, USA) equipped with a 1.9 µm C18 column. Samples were separated using a 3 min gradient from 5 to 8%, 42 min from 8 to 44%, 5 min from 44 to 60%, 7 min from 60 to 100%, and 7 min maintained at 80% of Solvent B (80% acetonitrile, 0.1% formic acid) at 200 nL/min. Separated peptides were analyzed using a tandem mass spectrometer (Orbitrap Exploris 480, Thermo Fisher Scientific, San Jose, CA, USA) with its parameters set as a data-dependent acquisition mode with a full MS scan (350–1600 *m/z* range) at a resolution of 60,000, followed by a full MS2 scan (100 *m*/*z*) at a resolution of 15,000, charge of 2+ to 7+, and dynamic exclusion setting of 30 s.

### 4.10. TMT Protein Identification and Quantification

Raw MS/MS spectrum data were processed with Thermo Proteome Discoverer 1.4.1.14 (Thermo Fisher Scientific, Waltham, MA, USA) and searched with the MASCOT search engine (Matrix Science, London, UK; version 2.3.02) against the *K. pneumoniae* database (NCBI: txid573). The peptide identification was based on a mass tolerance of 20 ppm for intact peptide masses and a mass tolerance of 0.05 Da for fragmented ions. Carbamidomethyl (C), TMT10plex (N-term), and TMT10plex (K) represent fixed modifications, and Oxidation (M), Deamidated (NQ), and TMT10plex (Y) represent variable modifications. All unique peptides (at least one unique spectrum) were required for protein identification and quantification. Then, labeled peptides were quantitatively analyzed with the IQuant software [62] using the Mascot Percolator algorithm [63] in both the peptide spectrum match (PSM) and protein- level with the chosen protein false discovery rate (FDR) strategy (FDR = 1%) [64]. The significant DEPs between the comparison groups were identified by fold change of >2 and *p*-values < 0.05.

### 4.11. Bioinformatics and Statistical Analysis

The GO and KEGG databases were used for functional and pathway annotations of all identified proteins. Enrichment analyses for the GO and KEGG pathways were carried out by using the hypergeometric distribution method to explore the biological functions and enrichment pathways of the DEPs between the compared samples. Entries with *p*-values < 0.05 were considered significant. The DEPs identified from the proteomic results were mapped onto the Search Tool for the Retrieval of Interaction Genes/Proteins (STRING) database (http://string-db.org/, accessed on 1 May 2022) to develop a PPI network (confidence score > 0.7). The Cytoscape software was used to visualize the PPI network.

## Figures and Tables

**Table 1:** Antimicrobial susceptibility of the DS, MDR, and XDR isolates.

Antibiotics	MIC of Strains (μg/mL)								
	DS-1	DS-2	DS-3	MDR-1	MDR-2	MDR-3	XDR-1	XDR-2	XDR-3
CST	≤0.5	≤0.5	≤0.5	≤0.5	≤0.5	≤0.5	**32**	**>64**	**>64**
AMK	≤2	≤2	≤2	**≥64**	**≥64**	**≥64**	**≥64**	**≥64**	**≥64**
GEN	≤1	≤1	≤1	**≥16**	**≥16**	**≥16**	**≥16**	**≥16**	**≥16**
TOB	≤1	≤1	≤1	**≥16**	**≥16**	**≥16**	**≥16**	**≥16**	**≥16**
IPM	≤1	≤1	≤1	**≥16**	**≥16**	**≥16**	**≥16**	**≥16**	**≥16**
MEM	≤0.25	≤0.25	≤0.25	**≥16**	**≥16**	**≥16**	**≥16**	**≥16**	**≥16**
CAZ	≤1	≤1	≤1	**≥64**	**≥64**	**≥64**	**≥64**	**≥64**	**≥64**
CRO	≤1	≤1	≤1	**≥64**	**≥64**	**≥64**	**≥64**	**≥64**	**≥64**
FEP	≤1	≤1	≤1	**≥32**	**≥32**	**≥32**	**≥32**	**≥32**	**≥32**
TZP	≤4	≤4	≤4	**≥128**	**≥128**	**≥128**	**≥128**	**≥128**	**≥128**
AZM	≤1	≤1	≤1	**≥64**	**≥64**	**≥64**	**≥64**	**≥64**	**≥64**
CIP	≤0.25	≤0.25	≤0.25	**≥4**	**≥4**	**≥4**	**≥4**	**≥4**	**≥4**
LVX	≤0.25	≤0.25	≤0.25	**≥8**	**≥8**	**≥8**	**≥8**	**≥8**	**≥8**
SXT	≤20	≤20	≤20	40	≤20	≤20	≤20	≤20	≤20
SFP	≤16	≤16	≤16	**≥64**	**≥64**	**≥64**	**≥64**	**≥64**	**≥64**
TGC	2	≤1	≤1	2	≤1	≤1	2	≤1	2

CST, Colistin; AMK, Amikacin; GEN, Gentamicin; TOB, Tobramycin; IPM, Imipenem; MEM, Meropenem; CAZ, Ceftazidime; CRO, Ceftriaxone; FEP, Cefepime; TZP, Piperacillin/Tazobactam; AZM, Aztreonam; CIP, Ciprofloxacin; LVX, Levofloxacin; SXT, Trimethoprim/Sulfa; SFP, Cefoperazone/Sulbactam; TGC, Tigecycline. Resistance is emphasized in bold.

**Table 2:** MLST and molecular characteristics of the DS, MDR, and XDR isolates.

Isolate	Housekeeping Genes							ST	Carbapenemases	Virulence Genes	String Test	Biofilm Formation (OD590 nm)
	*gapA*	*infB*	*mdh*	*pgi*	*phoE*	*rpoB*	*tonB*					
DS1	2	2	1	1	3	3	3	ST5	-	*iucA*, *iroB*, *peg-344*	-	0.2264
DS2	18	15	26	108	32	37	51	ST1304	-	*iucA*, *peg-344*	-	0.5713
DS3	2	1	1	1	9	4	12	ST23	-	*iucA*, *iroB*, *peg-344*, *_p_rmpA*, *_p_rmpA2*	+	0.2334
MDR1	3	3	1	1	1	1	4	ST11	KPC	*iucA*, *peg-344*, *_p_rmpA2*	+	0.28933
MDR2	3	3	1	1	1	1	4	ST11	KPC	*iucA*, *iroB*, *peg-344*, *_p_rmpA*, *_p_rmpA2*	+	0.36367
MDR3	3	3	1	1	1	1	4	ST11	KPC	*iucA*, *peg-344*, *_p_rmpA2*	+	0.15033
XDR1	3	3	1	1	1	1	4	ST11	KPC	*iucA*, *iroB*, *peg-344*, *_p_rmpA2*	-	0.76367
XDR2	3	3	1	1	1	1	4	ST11	KPC	*iucA*, *peg-344*, *_p_rmpA2*	-	0.41033
XDR3	3	3	1	1	1	1	4	ST11	KPC	*iucA*, *peg-344*	-	0.61

## Data Availability

The data used to support the findings of this study are available from the corresponding author upon request.

## Supplementary Materials

The following are available online at https://www.mdpi.com/article/10.3390/antibiotics11101341/s1, Table S1:The details of the DEPs between the MDR and DS strains; Table S2: The details of the DEPs between the XDR and DS strains; Table S3: The details of the DEPs between the MDR and XDR strains; Table S4: GO function analysis of the DEPs between the MDR and DS strains; Table S5: KEGG pathway analysis of the DEPs between the MDR and DS strains; Table S6: GO function analysis of the DEPs between the XDR and DS strains; Table S7: KEGG pathway analysis of the DEPs between the XDR and DS strains; Table S8: GO function analysis of the DEPs between the MDR and XDR strains; Table S9: KEGG pathway analysis of the DEPs between the MDR and XDR strains; Table S10: The sequences of primers used in this study [18,55,56]; Table S11: TMT labeling information of the DS, MDR, and XDR strains.
