# Supplementary material for: Molecular Characteristics and Quantitative Proteomic Analysis of Klebsiella pneumoniae Strains with Carbapenem and Colistin Resistance
Source: Antibiotics (Basel). 2022 Sep 30;11(10):1341. doi: 10.3390/antibiotics11101341 (PMC9598126; doi:10.3390/antibiotics11101341)
Supplement: Supplementary file 1 [file antibiotics-11-01341-s001.zip › antibiotics-1931230-supplementary/Table S10.pdf]

Table S10: The sequences of primers used in this study.

Primer	Nucleotide Sequence (5′ to 3′)	Target gene	Fragment (bp)	Refrences
mcr1-F	ATCAGCCAAACCTATCCCATCG	*mcr-1*	1257	[55]
mcr1-R	GCAGACGCACAGCAATGCCTAT			
mcr2-F	GCGATGGCGGTCTATCCTGTAT	*mcr-2*	378	[55]
mcr2-R	TGCGATGACATGGGGTGTCAGC			
mcr3-F	TATGGGTTACTATTGCTGG	*mcr-3*	814	[55]
mcr3-R	CTACCCTGATGCTCATCG			
mcr4-F	GTCATAGTGGTATAAAAGTACAG	*mcr-4*	669	[55]
mcr4-R	CCACCGTCTATCAGAGCCAAC			
mcr5-F	GCGGTTGTCTGCATTTATCAC	*mcr-5*	1042	[55]
mcr5-R	CTTTGAAAACCTGTCTTCGGCA			
mcr6-F	GTCCGGTCAATCCCTATCTGT	*mcr-6*	556	[55]
mcr6-R	ATCACGGGATTGACATAGCTAC			
mcr7-F	TGCTCAAGCCCTTCTTTTCGT	*mcr-7*	892	[55]
mcr7-R	TTCATCTGCGCCACCTCGT			
mcr8-F	AACCGCCAGAGCACAGAATT	*mcr-8*	667	[55]
mcr8-R	TTCCCCCAGCGATTCTCCAT			
KPC-F	CGTCTAGTTCTGCTGTCTTG	bla_{KPC}	798	[56]
KPC-R	CTTGTCATCCTTGTTAGGCG			
NDM-F	GGTTTGGCGATCTGGTTTTC	bla_{NDM}	621	[56]
NDM-R	CGGAATGGCTCATCACGATC			

OXA48-F	GCGTGGTTAAGGATGAACAC	*bla*$_{OXA-48}$	438	[56]
OXA48-R	CATCAAGTTCAACCCAACCG			
IMP-F	GGAATAGAGTGGCTTAAYTCTC	*bla*$_{IMP}$	232	[56]
IMP-R	GGTTTAAYAAAACAACCACC			
VIM-F	GATGGTGTTTGGTCGCATA	*bla*$_{VIM}$	390	[56]
VIM-R	CGAATGCGCAGCACCAG			
iucA-F	GCTTATTTCTCCCCAACCC	*iucA*	583	[18]
iucA-R	TCAGCCCTTTAGCGACAAG			
iroB-F	CAAAAAAGCAGCAGAGGC	*iroB*	585	[18]
iroB-R	TCACTGGCGGAATCCAACAC			
peg344-F	AAAGGACAGAAAGCCAGTG	*peg-344*	411	[18]
peg344-R	CAATGACGAGGGGGATAATC			
rmpA-F	GAGTAGTTAATAAATCAATAGCAAT	$_{p}$*rmpA*	332	[18]
rmpA-R	CAGTAGGCATTGCAGCA			
rmpA2-F	GTGCAATAAGGATGTTACATTA	$_{p}$*rmpA2*	430	[18]
rmpA2-R	GGATGCCCTCCTCCTG			

F, sense primer; R, antisense primer.
