# Supplementary material for: Molecular Characteristics and Quantitative Proteomic Analysis of Klebsiella pneumoniae Strains with Carbapenem and Colistin Resistance
Source: Antibiotics (Basel). 2022 Sep 30;11(10):1341. doi: 10.3390/antibiotics11101341 (PMC9598126; doi:10.3390/antibiotics11101341)
Supplement: Supplementary file 1 [file antibiotics-11-01341-s001.zip › antibiotics-1931230-supplementary/Table S11.pdf]

Table S11: TMT labeling information of the DS, MDR and XDR strains.

**Group**	**Sample**	**TMT isotope**
DS	DS1	126
	DS2	127N
	DS3	127C
MDR	MDR1	128N
	MDR2	128C
	MDR3	129N
XDR	XDR1	129C
	XDR2	130N
	XDR3	130C
